# O Novo Paradigma da Mensuração da Pressão Arterial

**DOI:** 10.36660/abc.20210592

**Published:** 2021-09-01

**Affiliations:** 1 Faculdade de Ciências Médicas de Minas Gerais Belo Horizonte MG Brasil Faculdade de Ciências Médicas de Minas Gerais, Belo Horizonte, MG - Brasil; 2 Instituto de Hipertensão Arterial - Diretoria Clínica Belo Horizonte MG Brasil Instituto de Hipertensão Arterial - Diretoria Clínica, Belo Horizonte, MG - Brasil

**Keywords:** Doenças Cardiovasculares/mortalidade, Pressão Arterial, Hipertensão, Monitorização Ambulatorial da Pressão Arterial(MAPA)/ métodos, Telemedicina, Metanálise

A hipertensão arterial (HA) é o principal fator de risco evitável para doenças cardiovasculares (DCV) e mortalidade por todas as causas em todo o mundo.^1^^,^^2^

A precisão na mensuração da pressão arterial (PA) é essencial para o diagnóstico, a estratificação de risco e o adequado tratamento da HA. Evidências demonstram que a tradicional avaliação casual da PA no consultório médico não é, na maioria dos casos, a melhor ferramenta para o diagnóstico, assim como para a tomada de decisão clínica em indivíduos com PA elevada ou mesmo no seguimento do tratamento da HA.^3^^,^^4^

Recentes diretrizes têm preconizado o uso mais amplo das medidas ambulatoriais e residenciais da PA na avaliação inicial de pacientes com PA elevada, assim como no acompanhamento de pacientes hipertensos.^4^^–^^8^ Medidas ambulatoriais e domiciliares da PA têm se revelado estratégias eficazes para detectar fenótipos intermediários confundidores comuns, como a hipertensão do avental branco e a hipertensão mascarada, não bem evidenciadas na medição usualmente realizada no consultório. Ademais, as monitorizações realizadas no ambiente habitual de cada indivíduo têm se correlacionado mais fortemente com o risco de desfechos clínicos maiores e lesões de órgãos-alvo.^3^^,^^4^^,^^9^^,^^10^

Na literatura e prática médica clínica internacional, são descritas duas modalidades de avaliação da PA fora do consultório: a monitorização ambulatorial da pressão arterial (MAPA), em que o equipamento é programado para realizar medidas automáticas em intervalos programados nas 24 horas; e a medição domiciliar da PA, em que cada indivíduo realiza as medidas livremente em horários determinados ou não, e os valores são registrados manualmente. No Brasil, embora realizemos a MAPA como em demais países, a medição domiciliar da PA é usualmente feita de duas maneiras distintas. Uma delas é a forma não estruturada, que denominamos de automedida da PA (AMPA), e a outra é uma forma peculiar de registro, comum em nosso meio, que é a medida residencial da PA (MRPA), em que equipamentos semiautomáticos armazenam as medições e, segundo protocolo estabelecido em diretrizes, são capazes de emitir laudos estruturados.^3^^,^^4^

Esta edição dos Arquivos Brasileiros de Cardiologia traz o artigo original “Hipertensos Tratados e Avaliados por Telemonitoramento Residencial da Pressão Arterial. Estudo TeleMRPA”, de Barroso et al.,^11^ que avalia os achados de um banco de dados formado por exames realizados em diferentes localidades brasileiras e reunidos em uma plataforma central de análise a distância por telemedicina.^11^

Embora não existam estudos comparativos entre AMPA e MRPA, estima-se que o método estruturado (MRPA) seja mais reprodutível, embora a avaliação livre (AMPA) tenha os benefícios do melhor custo e possibilidade de ser realizado habitual e frequentemente como aliado do seguimento.^3^ Há também diferenças sensíveis entre MRPA e MAPA. A MRPA é realizada na posição sentada e em casa, enquanto a MAPA é realizada em condições diversas, como em casa ou no trabalho, durante atividades diárias de rotina e sem um período de descanso sentado antes das medições. Além disso, na MAPA é possível analisar a PA durante o sono, informação que agrega maior valor prognóstico ao método.^4^ Valores médios da vigília, contudo, foram considerados comparáveis entre aqueles avaliados por medidas residenciais e pela MAPA^4^. Há evidências de que valores pressóricos médios elevados em um dos dois métodos (seja na MAPA ou na MRPA) estejam associados a maior risco cardiovascular (RCV) que a normotensão evidenciada em ambos. No entanto, quando houve elevação pressórica média em ambos os métodos, o RCV foi ainda maior. Assim, os dois métodos devem ser considerados não como técnicas competitivas, mas como complementares na avaliação da PA.^12^^,^^13^

Um estudo realizado em Ohasama, no Japão, revelou originalmente que a PA domiciliar tinha maior valor preditivo para mortalidade do que a PA casual de consultório na população geral.^14^ Estudos prospectivos comprovaram a superioridade das medidas residenciais sobre a PA casual na predição de RCV.^14^ Uma metanálise reforçou as evidências de que a PA residencial seria um melhor preditor de DCV e morte cardiovascular, permitindo uma estratificação mais precisa de RCV do que a PA casual, particularmente nos casos de hipertensão mascarada.^15^ Considerando os desfechos intermediários, uma metanálise mostrou que a PA residencial elevada se correlacionou mais significativamente com danos subclínicos em órgãos-alvo, avaliada principalmente pelo índice de massa ventricular esquerda ao ecocardiograma, quando comparada com a PA casual, tendo valor semelhante à MAPA.^16^

Considerando a maior precisão diagnóstica e sua capacidade prognóstica, a PA residencial desempenha também um importante papel no seguimento do tratamento da HA, pois é superior à PA casual em condições como o efeito do avental branco e a hipertensão não controlada mascarada em pacientes tratados, assim como na titulação de fármacos.^17^ Além disso, a MRPA resulta em menor restrição à realização de atividades diárias e menor desconforto no sono que a MAPA.^3^^,^^4^ Há também evidências de que o emprego de PA residencial associada ao telemonitoramento remoto foi superior às medições de PA casual para o controle terapêutico de pacientes hipertensos.^18^

Diante de tantas restrições à medida casual e na tentativa de apurar a acurácia da medição da PA no consultório, o estudo *Systolic Blood Pressure Intervention Trial* (SPRINT) utilizou uma metodologia que incluía um período de repouso prévio e adotou a média de múltiplas leituras em cada visita. O protocolo incluía ainda uso de manguito de tamanho apropriado, posicionamento adequado do paciente, apoio para as costas, pés apoiados no chão, braço apoiado no nível do coração e manguito instalado em braço nu, com as medições realizadas na forma de automedições sob vigilância dos pesquisadores.^19^ Embora tenham surgido argumentações de que a técnica de mensuração da PA no SPRINT seria atípica, esse protocolo seguiu as recomendações da maioria das diretrizes, as quais foram semelhantes às adotadas em muitos ensaios clínicos anteriores, além de servir de modelo para estudos posteriores e reforçar orientações quanto à mais adequada técnica de medição da PA no consultório.^19^

No estudo de Barroso et al.,^11^ que avaliou os valores de MRPA por telemonitoramento de 6.731 hipertensos em tratamento farmacológico, sendo 61,3% do sexo feminino, com idade média de 57,8 (±12,6) anos e índice de massa corporal médio de 29,0 (±5,1) kg/m^2^, observou-se que os valores médios de PA sistólica foram 6,6 mmHg (p<0,001) e de PA diastólica, 4,4 mmHg (p<0,001) maiores que na medida casual. Também, comparativamente, a taxa de controle da HA foi de 61,3% pela MRPA, sendo este percentual maior que o encontrado na medida casual (57,0%, p<0,001). O estudo demonstrou ainda uma prevalência de efeito avental branco de 15,4% e de hipertensão mascarada não controlada de 11,1% da população avaliada.^11^

Uma publicação anterior preliminar do mesmo banco de dados, envolvendo 1.273 participantes com pré-hipertensão e HA estágio 1, havia encontrado hipertensão do avental branco em 21,9% e hipertensão mascarada em 11,4%, além de discordância com o diagnóstico de HA por PA casual em 33,3% pacientes^20^ Ao revelar a realidade da MRPA em nosso meio, o estudo traz importante contribuição ao conjunto de evidências que demonstram os benefícios do método em avaliar o tratamento da HA, reforçando a necessidade de uma utilização mais ampla da ferramenta no seguimento do hipertenso.

Diante das evidências da importância do monitoramento da PA, é necessário refletir sobre os emergentes dispositivos de medição da PA fora do consultório, muitos dos quais são vestíveis, ofertando possibilidades de ilimitados registros, além de armazenamento e de análises digitais. Novos monitores digitais, agora disponíveis até mesmo sem a necessidade do uso de braçadeiras, oferecem maior comodidade e já vêm sendo empregados em ensaios clínicos randomizados, embora muitos ainda estejam em processo de validação. Além disso, o advento da inteligência artificial amplia as possibilidades de análise desse grande número de registros de PA fornecidos por tais dispositivos, que, em conjunto com outras variáveis, propiciarão uma verdadeira revolução do conhecimento acerca da PA em um futuro muito próximo.^21^
