# Supplementary material for: O Novo Paradigma da Mensuração da Pressão Arterial
Source: Arq Bras Cardiol. 2021 Sep 1;117(3):528–30. [Article in Portuguese] doi: 10.36660/abc.20210592 (PMC8462964; doi:10.36660/abc.20210592)
Supplement: Translation

# Translation: The New Paradigm of Blood Pressure Measurement

**Affiliations:** 1 Belo Horizonte MG Brazil Faculdade de Ciências Médicas de Minas Gerais, Belo Horizonte, MG - Brazil; 2 Belo Horizonte MG Brazil Instituto de Hipertensão Arterial - Diretoria Clínica, Belo Horizonte, MG - Brazil

**Keywords:** Cardiovascular Diseases/mortality, Blood Pressure, Hypertension, Blood Pressure Monitoring,Ambulatory/methods, Telemedicine, Meta-Analysis

Hypertension (HTN) is the leading preventable risk factor for cardiovascular disease (CVD) and all-cause mortality worldwide.^1^^,^^2^

Accuracy in blood pressure (BP) measurement is essential for the diagnosis, risk stratification, and adequate treatment of HTN. The evidence shows that the traditional casual assessment of BP in the doctor’s office is not, in most cases, the best tool for diagnosis or clinical decision-making in individuals with high BP, or even for monitoring response to antihypertensive treatment.^3^^,^^4^

Recent guidelines have advocated the broader use of outpatient and home BP measurements in the initial assessment of patients with high BP, as well as in the follow-up of patients with known HTN.^4^^–^^8^ Outpatient and home BP measurements have been shown to be effective strategies to detect common confounding intermediate phenotypes, such as white coat hypertension and masked hypertension, which are poorly demonstrated by office measurements. Furthermore, monitoring BP in the patient’s usual environment has been found to correlated more strongly with the risk of major clinical outcomes and target organ damage.^3^^,^^4^^,^^9^^,^^10^

In the literature and international clinical medical practice, two broad types of out-of-office BP assessment are described: ambulatory blood pressure monitoring (ABPM), in which the device is programmed to obtain automatic measurements at preset intervals over a 24-hour period; and home BP measurement, in which patients themselves obtain measurements at predetermined or random times and BP values are manually recorded. In Brazil, although ABPM is done as in other countries, household BP measurement is usually done in two different ways: one unstructured, which we call self-measurement of BP (SMBP), and a peculiar form of recording quite common in our country, home blood pressure monitoring (HBPM), based on the use of semi-automatic sphygmomanometers which stores measurements and, according to a protocol defined in clinical guidelines, must be capable of issuing structured reports.^3^^,^^4^

This issue of ABC Cardiol carries the original article “Treated Hypertensive Patients Assessed by Home Blood Pressure Telemonitoring. TeleMRPA Study”, by Barroso et al.,^11^ which assesses the findings of a database of measurements performed in different Brazilian locations and collected in a central platform for remote analysis via telemedicine.^11^

Although there are no head-to-head comparisons of SMBP and HBPM, the structured method (HBPM) is believed to be more reproducible, although freeform measurement (SMBP) is less costly and can be performed habitually and frequently as an adjunct to follow-up.^3^ There are also substantial differences between HBPM and ABPM. HBPM is performed by patients at home, in the seated position, while ABPM is performed under a variety of conditions at home or at work, during routine activities of daily living, and with no periods of rest prior to each measurements. In addition, ABPM allows analysis of overnight BP during sleep, which adds greater prognostic value to this method.^4^ Average waking BP values, however, have considered comparable whether measured by home-based methods or by ABPM^4^. There is evidence that elevated mean BP values in either one of the two methods (either ABPM or HBPM) is associated with greater cardiovascular risk (CVR) than normal values evidenced by both methods. However, when mean BP was elevated in both ABPM and HBPM, the CVR was even higher. Thus, the two methods should be considered not as competitive, but as complementary techniques for BP assessment.^12^^,^^13^

A study carried out in Ohasama, Japan, originally revealed that home BP had greater predictive value for mortality than casual BP in the general population.^14^ Prospective studies have since proven the superiority of home measurements over casual BP in predicting CVR.^14^ A meta-analysis provided further evidence that home BP may be a better predictor of CVD and cardiovascular death, allowing for a more accurate stratification of CVR than casual BP measurement, particularly in cases of masked hypertension.^15^ Considering surrogate endpoints, a meta-analysis showed that elevated home BP measurements correlated more significantly with subclinical target organ damage, assessed mainly by the left ventricular mass index on echocardiography, when compared to casual BP, having a predictive value similar to that of MAP.^16^

In view of its greater diagnostic accuracy and prognostic capacity, home BP measurement also plays an important role in the follow-up of antihypertensive treatment, as it is superior to casual BP in conditions such as white coat hypertension and masked uncontrolled hypertension in treated patients, as well as for monitoring response to drug titration.^17^ In addition, HBPM imposes fewer restrictions on daily activities and is more comfortable during sleep than ABPM.^3^^,^^4^ There is also evidence that the combination of home BP measurement and remote telemonitoring was superior to casual BP measurement for treatment control of patients with known hypertension.^18^

Given these many restrictions on casual measurement and in an attempt to ascertain the accuracy of office BP measurement, the *Systolic Blood Pressure Intervention Trial* (SPRINT) used a method that included a rest period before each measurement and averaged multiple readings obtained at each visit for analysis. The protocol also included the use of an appropriately sized cuff, proper patient positioning, back support, feet flat on the floor, arm supported at heart level and cuff placed on a bare arm, with self-measurement performed under the supervision of the investigators.^19^ Although arguments have emerged that the BP measurement technique used in the SPRINT trial is atypical, this protocol followed the recommendations of most clinical practice guidelines and was similar to that adopted in many previous trials, in addition to serving as a model for future studies and strengthening guidance regarding the most appropriate technique for office BP measurement.^19^

In the study by Barroso et al.,^11^ who used telemonitoring to evaluate HBPM values of 6,731 patients with hypertension undergoing pharmacological treatment (61.3% female; mean age 57.8±12.6 years; mean BMI 29.0±5.1 kg/m^2^), mean systolic BP values were 6.6 mmHg (p<0.001) higher and diastolic BP 4.4 mmHg higher (p<0.001) than on casual measurement. Comparatively, the prevalence of controlled HTN was 61.3% by HBPM versus 57.0% by casual BP measurement (p<0.001). The study also showed a 15.4% prevalence of the white coat effect and 11.1% prevalence of uncontrolled masked hypertension in the evaluated population.^11^

A previously published interim analysis of the same database, involving 1273 participants with prehypertension and stage 1 HTN, had found white coat hypertension in 21.9% and masked hypertension in 11.4%, as well as disagreement with the diagnosis of hypertension by casual BP measurement in 33.3% of patients.^20^

By revealing the reality of HBPM in our country, this study makes an important contribution to the body of evidence that demonstrates the benefits of this method in evaluating the response to HTN treatment, highlighting the need for broader use of this tool in the follow-up of hypertensive patients.

Given the evidence of the importance of monitoring of BP, it is necessary to reflect on the emerging devices of BP measurement outside the office, many of which are wearable, providing the possibility of unlimited recording, storage, and digital analysis. New digital monitors, which now even dispense the use of inflatable armbands or cuffs, offer greater convenience, and although many have yet to be validated, these are already being used in randomized clinical trials. In addition, the advent of artificial intelligence expands the possibilities of analysis of the enormous body of BP records provided by such devices, which, together with other variables, should bring about a true revolution in knowledge about HTN in the very near future.^21^
